# Offspring's Leukocyte Telomere Length, Paternal Age, and Telomere Elongation in Sperm

**DOI:** 10.1371/journal.pgen.0040037

**Published:** 2008-02-15

**Authors:** Masayuki Kimura, Lynn F Cherkas, Bernet S Kato, Serkalem Demissie, Jacob B Hjelmborg, Michael Brimacombe, Adrienne Cupples, Janice L Hunkin, Jefferey P Gardner, Xiaobin Lu, Xiaojian Cao, Malinee Sastrasinh, Michael A Province, Steven C Hunt, Kaare Christensen, Daniel Levy, Tim D Spector, Abraham Aviv

**Affiliations:** 1 The Center of Human Development and Aging, University of Medicine and Dentistry of New Jersey, New Jersey Medical School, Newark, New Jeresey, United States of America; 2 Twin Research and Genetic Epidemiology Unit, St. Thomas' Hospital Campus, Kings College London, London, United Kingdom; 3 Boston University School of Public Health, Department of Biostatistics, Boston, Massachusetts, United States of America; 4 The Institute of Public Health, Epidemiology and Statistics, University of Southern Denmark, Odense, Denmark; 5 Division of Statistical Genomics, Washington University School of Medicine, St. Louis, Missouri, United States of America; 6 Cardiovascular Genetics, University of Utah School of Medicine, Salt Lake City, Utah, United States of America; 7 The National Heart, Lung, and Blood Institute's Framingham Heart Study, Boston, Massachusetts, United States of America; National Institute of Genetics, Japan

## Abstract

Leukocyte telomere length (LTL) is a complex genetic trait. It shortens with age and is associated with a host of aging-related disorders. Recent studies have observed that offspring of older fathers have longer LTLs. We explored the relation between paternal age and offspring's LTLs in 4 different cohorts. Moreover, we examined the potential cause of the paternal age on offspring's LTL by delineating telomere parameters in sperm donors. We measured LTL by Southern blots in Caucasian men and women (n=3365), aged 18–94 years, from the Offspring of the Framingham Heart Study (Framingham Offspring), the NHLBI Family Heart Study (NHLBI-Heart), the Longitudinal Study of Aging Danish Twins (Danish Twins), and the UK Adult Twin Registry (UK Twins). Using Southern blots, Q-FISH, and flow-FISH, we also measured telomere parameters in sperm from 46 young (<30 years) and older (>50 years) donors. Paternal age had an independent effect, expressed by a longer LTL in males of the Framingham Offspring and Danish Twins, males and females of the NHLBI-Heart, and females of UK Twins. For every additional year of paternal age, LTL in offspring increased at a magnitude ranging from half to more than twice of the annual attrition in LTL with age. Moreover, sperm telomere length analyses were compatible with the emergence in older men of a subset of sperm with elongated telomeres. Paternal age exerts a considerable effect on the offspring's LTL, a phenomenon which might relate to telomere elongation in sperm from older men. The implications of this effect deserve detailed study.

## Author Summary

Leukocyte telomere length becomes shorter with age and is apparently a biomarker of aging and a forecaster of longevity in humans. Leukocyte telomere length is heritable, longer in women than in men, and is relatively shorter in persons who suffer from aging-related diseases, cardiovascular diseases in particular. This study found in four different populations that leukocyte telomere length in adult offspring was positively correlated with paternal age at the time of birth of the offspring. Analysis of telomeres in sperm of young (<30 years) and older (>50 years) donors revealed the emergence in the older donors of a subset of sperm with elongated telomeres. The mechanisms behind this enigmatic, age-dependent elongation in telomere length of sperm are unknown but may relate to epigenetic factors or the survival of a subset of germ-line stem cells, resilient against aging. It is also unknown how older fathers endow their offspring with longer telomeres in their leukocytes. The potential impact of paternal age on leukocyte telomere length and, conceivably, aging-related diseases and longevity in the offspring is of relevance because offspring of older fathers comprise an increasing proportion of society.

## Introduction

Leukocyte telomere length (LTL) is a complex genetic trait. Though highly variable, it is heritable [1–6] and longer in women than men [1,3,4,6–9]. Environmental factors, including smoking [3,10], obesity [9–11], psychological stress [12] and low socio-economic status (SES) [13] are ostensibly associated with shortened LTL, underscoring the roles of not only genetic factors but also the environment in fashioning leukocyte telomere dynamics (length and attrition rate). Shortened LTL is also observed in individuals with aging-related diseases, including hypertension [1,7], insulin resistance [11,14,15], atherosclerosis [16,17], myocardial infarction [16,18,19], stroke [9] and dementia [20,21]. Further, aging itself may modify the relationship between LTL and some of these variables [reviewed 22].

Although the mechanisms that account for variations among humans in LTL are not fully understood, increased oxidative stress and inflammation – two potential determinants of life span and aging-related diseases [23,24] – are likely to heighten age-dependent telomere attrition in leukocytes [9,12,14, 25 reviewed in 26]. As aging-related diseases and environmental factors may cause premature mortality, and as men's LTL [1,7,9,14,25] and life expectancy [27] are shorter than women's, the potential relation between LTL and human longevity has been explored in several studies, which yielded conflicting results [18,28–31].

In light of these diverse observations, we need to further understand the biological determinants of LTL and their role in aging-related diseases. Two studies, comprising 125 [32] and 2,433 [33] participants observed a positive correlation between the LTL in adult offspring and paternal age at their birth (paternal age). Here we report a two-phase exploration of the dependency of LTL in the offspring on paternal age. First, we describe the details of this phenomenon in 4 samples of wide age range distributions: the Offspring of the Framingham Heart Study (Framingham Offspring), the National Heart Lung and Blood Institute Family Heart Study (NHLBI-Heart), the Longitudinal Study of Aging Danish Twins (Danish Twins), and the UK adult twin registry (UK Twins). Second, we present data suggesting the emergence of a subset of sperm with longer telomeres in older men. Such a process might explain the dependency of the offspring's LTL on paternal age.

## Results

### First Phase: Leukocyte Telomere Length (LTL)

#### General characteristics.

The ages of the offspring, LTLs, the ages of their parents and, if available, data about smoking, body mass index (BMI) and socio-economic status (SES) are displayed in Tables S1–S4.

While individuals from the Framingham Offspring, NHLBI-Heart and UK Twins had a wide age range (18–86 years), the Danish Twins were all elderly and their age distribution was limited (73–94 years). In all cohorts, fathers' and mothers' ages were highly correlated (r = 0.72–0.85, all p< 0.001).

#### Son's age-adjusted LTL and paternal age.

**Figure 1:**
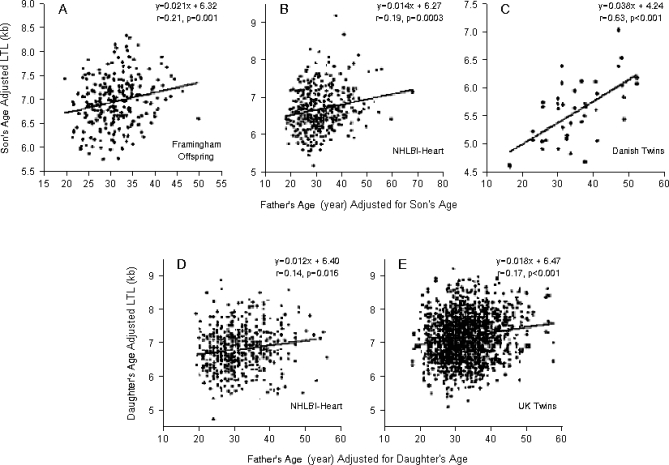
Offspring's Age-Adjusted LTL versus Father's Age at Birth of the Offspring, Adjusted for the Offspring's Age (A) Males (sons), Framingham Offspring; (B) Males (sons), NHLBI-Heart; (C) Males (sons), Danish Twins; (D) Females (daughters), NHLBI-Heart; (E) Females (daughters) UK Twins.

Three of the 4 cohorts included both male and female offspring. In these 3 cohorts, son's age-adjusted LTL was significantly correlated with paternal age (Figure 1, Tables 1–3). The strengths of these associations were: Framingham Offspring, r = 0.21, *p* = 0.001; NHLBI-Heart, r = 0.19, p = 0.0003; Danish Twins, r = 0.63, *p* < 0.001. These associations accounted for 4.5%, 3.6%, and 39.7% of the variations in the son's LTL for the Framingham Offspring, NHLBI-Heart and Danish Twins, respectively.

**Table 1:**
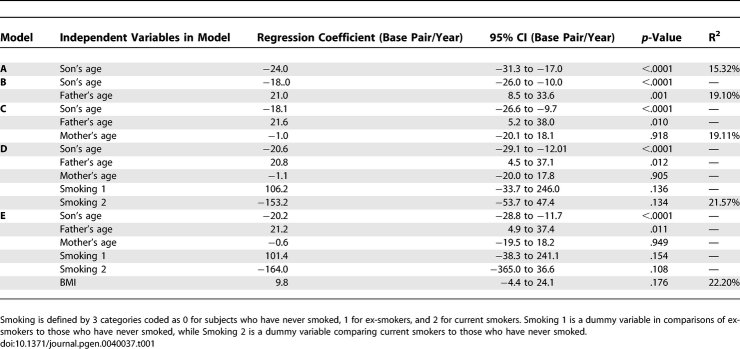
Regression of LTL on Parent's Age and Covariates in Sons of the Framingham Offspring

**Table 2:**
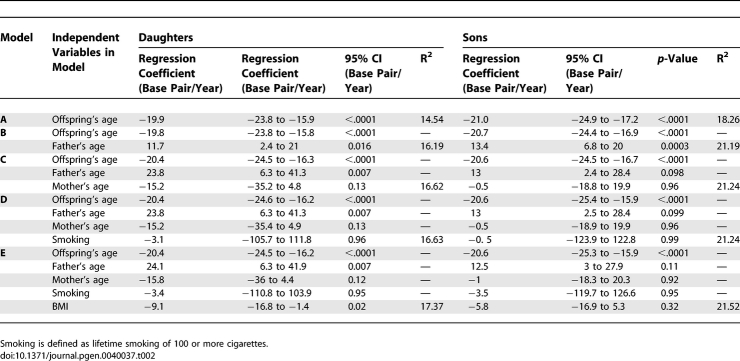
Regression of LTL on Parent's Age and Covariates in Offspring of the NHLBI-Heart

#### Daughter's age-adjusted LTL and paternal age.

All 4 cohorts included female offspring. In 2 of these cohorts, the NHLBI-Heart and UK Twins, daughter's age-adjusted LTL was significantly correlated with paternal age (Figure 1, Tables 2 and 4). The strengths of these associations were: NHLBI-Heart, r = 0.14, *p* = 0.016; UK Twins, r = 0.17, *p* < 0.001. These associations accounted for 1.9% and 3.0% of the variations in the daughter's LTL for the NHLBI-Heart and UK Twins, respectively. No significant associations were observed between the daughter's age-adjusted LTL and paternal age for the Framingham Offspring and Danish Twins.

**Table 3:**
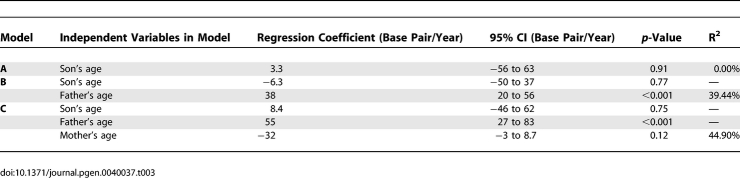
Regression of LTL on Parent's Age and Covariates in Sons of Danish Twins

**Table 4:**
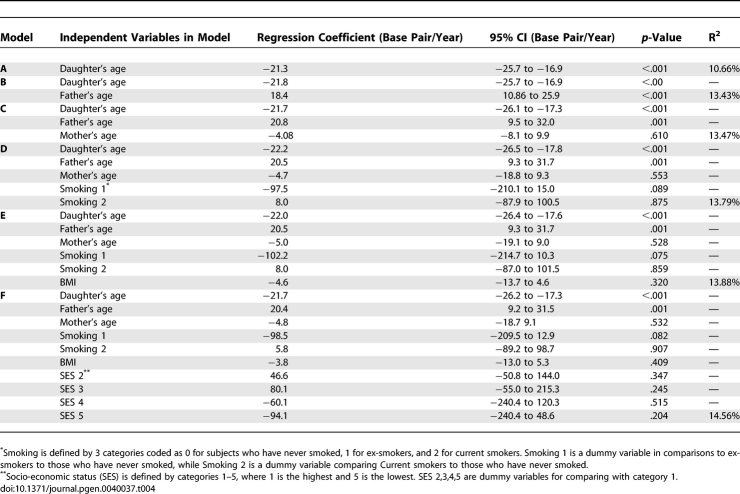
Regression of LTL on Parent's Age and Covariates in Daughters of Twins UK

#### Contribution of mother's age at the time of the offspring's birth (maternal age) to variations in the offspring's LTL.

As mother's and father's ages were highly correlated in all cohorts, we explored the contribution of maternal age to variations in the offspring whose age-adjusted LTL showed significant association with paternal age. Model C in Tables 1–4 displays the contribution of maternal age to the variation in the LTL, when considered jointly with the effect of a) the offspring's age, and b) paternal age. For the sons of the Framingham Offspring (Table 1) and Danish Twins (Table 3), the daughters of the NHLBI-Heart (Table 2) and UK Twins (Table 4), the inclusion of maternal age in the model did not diminish the significance of the independent contribution to the variation in the offspring's LTL by the paternal age. For the sons of the NHLBI-Heart (Table 2), the inclusion of maternal age in the model diminished the p value from 0.0003 to 0.098 for the contribution of paternal age to the variation in the son's LTL. In this cohort, the son's age-adjusted LTL was correlated not only with paternal age but also with maternal age at the time of birth of the son (r = 0.17, *p* = 0.004). Thus, when adjusting for the significant effect of the maternal age, the significance of the paternal age became only suggestive. However, the significant correlation between the son's age-adjusted LTL with maternal age became insignificant after adjusting for paternal age. Accordingly, paternal age was a major factor in the variation of the son's LTL.

#### Contribution of BMI, cigarette smoking, and SES to variation in the Offspring's LTL.

Whenever available, the contribution of these factors (models D, E, and F in Tables 1, 2, and 4) to the overall variation in the offspring's LTL was quite small.

UK Twins comprised the largest cohort, a subset of which (n = 1,000) had a complete data profile (Table 4), which did not differ from the entire cohort. In this group, the independent effect of paternal age on age-adjusted LTL in the daughters (r^2^ of 3.04%) was nearly twice as large in relative terms as other variables combined (BMI, smoking, SES, r^2^ = 1.58%).

#### The effect of the offspring's age versus paternal age on the offspring's LTL.

In the Framingham Offspring, LTL decreased at a rate of 24 base pairs (bp) per year (±3.7, SE; r = −0.21, *p* = 0.001) with son's age and at a rate of 18 bp per year (± 4.5, SE; r = −0.28, *p* < 0.001) with daughter's age. After adjustments for the age of the son and maternal age, the son's LTL had on average an additional 22 bp length for each year of the paternal age.

In the NHLBI-Heart, LTL decreased at a rate of 21 bp per year (± 2.0, SE; r = −0.43, *p* < 0.001) with son's age and at a rate of 20 bp per year (± 2.0, SE; r = −0.44, *p* < 0.001) with daughter's age. After adjustments for the ages of the offspring and maternal age, the son's and daughter's LTLs had on average additional 11 and 22 bp lengths, respectively for each year of paternal age.

In the Danish Twins, there was no significant decrease in the LTL with age in the sons. In the daughters, the LTL decreased with age at a rate of 21 bp per year (± 1.5, SE; r = −0.39, *p* < 0.001). After adjustments for the son's age and maternal age, the son's LTL had on average an additional 53 bp length for each year of paternal age.

In the UK Twins, LTL in the daughter decreased with age at a rate of 21 bp per year (± 1.5, SE; r = −0.39, *p* < 0.001). After adjustments for the daughter's age and maternal age, the daughter's LTL had on average an additional 23 bp length for each year of paternal age.

#### Summary of the principal observations of LTL.

Table 5 summarizes by gender the effect of the offspring's age versus paternal on the offspring's LTL.

**Table 5:**
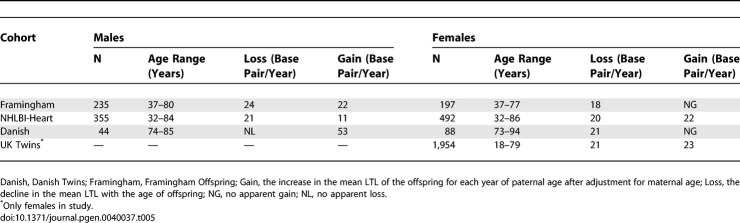
Summary of the Effect of Paternal Age on Offspring's LTL

### Second phase: Sperm telomere length

#### The effect of age on telomere length in sperm based on Southern blot analysis of the terminal restriction fragment length (TRFL).

**Figure 2:**
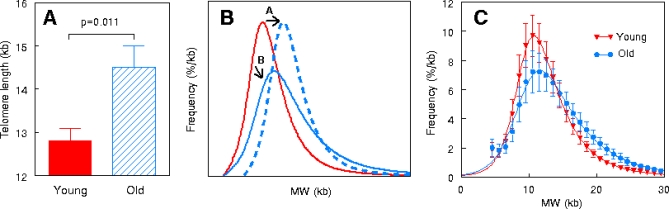
TRFL in Sperm from Young and Older Donors (A) Means of TRFLs in sperm of young and older donors. (B) Two hypothetical scenarios for TRFL distributions in sperm from young and older donors. Scenario A depicts a uniform shift in the TRFL distribution towards longer telomeres in sperm of older men (interrupted blue line) from that of younger men (red line). Scenario B depicts a reconfiguration of the distribution, so that shorter telomeres are disproportionably “under-represented,” while longer telomeres are “over-represented.” (C) Frequency distributions of TRFLs in sperm from young and older donors. For (C), data were fitted to a 4-parameter logistic dose response distribution curves, y = a_0_+a_1_/[1+(x/a_2_)^a3^], using TableCurve 2D software (SYSTAT Software).

In principle, the paternal age effect on the offspring's LTL might be related to sperm telomere length in the fathers. For this reason, we examined the effect of the donor's age on sperm TRFL in healthy volunteers. Figure 2A shows that sperm from older donors displayed a significantly longer mean TRFL than sperm from younger donors.

The TRFL is based on pooled DNA extracted from all sperm in each sample. It is derived from the distribution of telomeres of different lengths of the p and q arms of the 23 chromosomes in each sperm and millions of sperm in each sample. A longer TRFL in a sperm sample may arise from a number of scenarios: a uniform shift in the TRFL distribution towards longer telomeres (scenario A in Figure 2B); a reconfiguration of the distribution, so that shorter telomeres are disproportionably “under-represented,” while longer telomeres are “over-represented” (scenario B in Figure 2B), and a combination of scenarios A and B. Figure 2C displays a composite of the frequency distributions from which we derived the sperm TRFL data displayed in Figure 2A. The frequency distributions are more compatible with scenario B, in that they indicate that the longer TRFL in sperm from older donors was primarily due to “under-represented” shorter telomeres and “over-represented” longer telomeres.

#### The effect of age on telomere length in sperm based on quantitative (Q) fluorescence in situ hybridization (FISH) and flow-cytometric analysis (flow-FISH).

The TRFL analysis provided no information about whether in older donors the increased sperm TRFL was due to an increase in the length of telomeres within each sperm or an emergence of a subset of sperm with longer telomeres. To gain a further insight into this matter, we resorted to Q-FISH and flow-FISH analyses.

**Figure 3:**
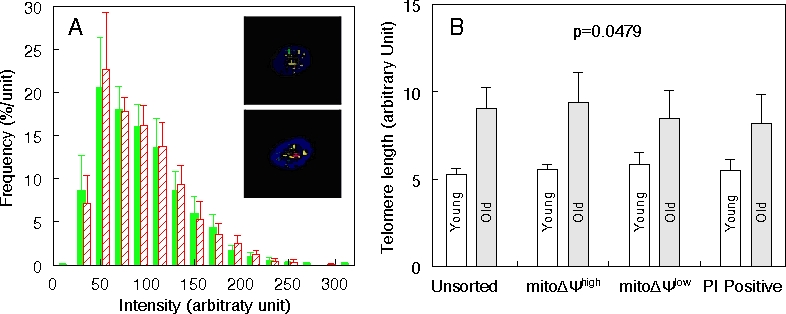
Telomere Signal Intensities by Q-FISH (A) Y and X sperm. Green, Y sperm; red, X sperm; insets display Y sperm (green) and X sperm (red); telomere signal is in yellow. (B) Unsorted and sorted sperm, based on mitochondrial membrane potential (MMP) determined by DiOC_6_(3) stain. H, high MMP; L, low MMP, Propidium iodide (PI) stained sperm are non-viable. Telomere length (*y*-axis) denotes the telomere signal intensity in sperm/telomere signal intensity in the reference chicken red blood cells.

We first examined by Q-FISH whether telomere length was different between X and Y sperm. Figure 3A shows that the telomere signal intensities were equivalent in both sperm types. Thus, we excluded a confounding effect of the X and Y sperm types on the telomere length in each sample. Next we examined by Q-FISH telomere length in subsets of sperm based on mitochondrial membrane potential (MMP), determined by 3,3′-dihexyoxacarbocyanine iodide (DiOC_6_(3)). We measured telomere signal intensity in both unsorted sperm and sorted sperm subsets, based on their DiOC_6_(3) staining (high, low). Telomere signal intensities were consistently higher in sperm from the older than younger donors, whether the sperm were unsorted or sorted according to the MMPs (Figure 3B).

**Figure 4:**
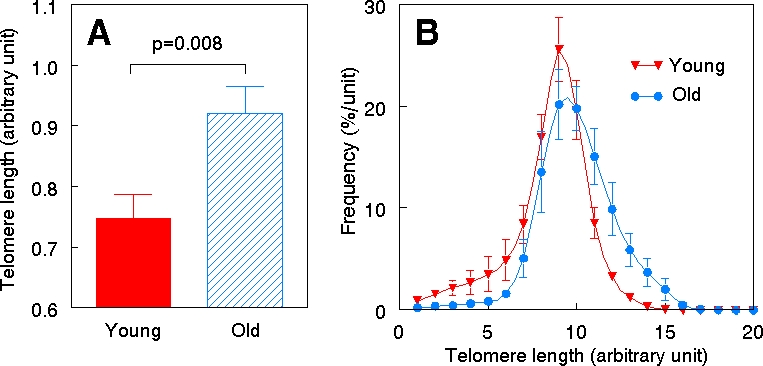
Telomere Lengths by Flow-FISH in Sperm from Young and Older Donors (A) Mean telomere length (in relative units) based on signal telomere intensity. (B) Composite of frequency distributions of signal intensity. Each bin represents 35 output signals. The connecting lines are spline estimations.

We then measured telomere length by flow-FISH in unsorted sperm from young and older donors. As shown in Figure 4, mean telomere length (in relative units), based on signal intensity, was longer in sperm from older than young donors (*p* = 0.008). To further explore the nature of the shift towards longer telomeres in sperm from older donors, we examined the distributions of young and older groups. These were non-normal distributions (Kolmogorov-Smirnov test, *p*-value < 0.01). As the distributions were non-normal we also examined the difference between sperm of older versus young donors in the median telomere length, which was significant (young donors 0.752 ± 0.038, older donors 0.884 ± 0.042, *p* = 0.031).

To assess the difference between the distributions, we chose a reasonable cut-off for flow-FISH response (500 events, i.e., 5%/unit on the *y*-axis of Figure 4B) and examined the proportion of values above the cut-off for each group. In order to assess the potential shift towards longer telomeres in the older group we examined the difference in proportions over the region marked as bin 12 – 15 on the *x*-axis of Figure 4B (sperm with relatively longer telomeres). Using the Fisher exact test, this difference was significant (*p*-value = 0.001), with a similar result for a cut-off at 7.5%/unit. For bin 1–7 on the *x*-axis of Figure 4B (sperm with relatively shorter telomeres), this difference was of a borderline significance (*p* = 0.065). Therefore, the data displayed in Figure 4B are consistent with over-representation of sperm with longer telomeres (and perhaps under-representation of sperm with shorter telomeres) in the older donors.

## Discussion

Unryn et al. [32] and more recently De Meyer et al. [33] observed that offspring's LTL was positively associated with paternal age. We confirmed this finding in leukocytes of 4 cohorts of wide age ranges from 3 countries. It is not clear why the offspring's LTL was positively correlated with paternal age in only males of the Framingham Offspring and in the Danish Twins. We suspect that in females a host of factors, including menopause [15,22], might confound associations between LTL and indices related to aging. Thus, much larger numbers of subjects might be needed to elucidate links between women's LTL and aging-related parameters. Indeed, in the present study, the effect of paternal age on the daughter's LTL was clearly observed in the NHLBI-Heart where sample size was more than 2 times and 5 times larger than those of the Framingham Offspring and the Danish Twins, respectively, and in the UK Twins, where sample size was about 10 and 20 times larger than those of the Framingham Offspring and Danish Twins, respectively.

When observed, the dependency of offspring's LTL on paternal age was considerable. This was particularly pronounced in the elderly males of the Danish Twins. Given that the elderly represent a selected group [22], it is possible that the dependency of offspring's LTL on paternal age is more expressed in those individuals who survived or resisted diseases of aging to reach old age. We also note that elderly males of the Danish Twins displayed no age-dependent LTL attrition. However, this was unlikely to be a feature of their old age. Rather, it probably related to the small cohort and its narrow age range (74–84 years; Table 5), which contrasted with the wide age ranges of the Framingham Offspring, the NHLBI-Heart and the UK Twins cohorts [22].

What are the potential factors that explain association between offspring's LTL and paternal age? The key might be age-dependent telomere dynamics in sperm. Mean telomere length in sperm does not decrease, and, in fact, increases with the donor's age [34,35], a phenomenon we confirmed. The donor's age effect on sperm telomere length was not due to the change in the sperm profile of MMP, a parameter serving as an indicator of sperm motility and ‘fertilizing' capacity [36]. Based on the flow-FISH analysis, the increase in sperm telomere length in older men appeared to arise from a subset of their sperm with longer telomeres. However, further studies in larger cohorts over a wide age span of donors are needed to confirm our conclusions and explore their meaning.

While a female is born with all the eggs she will have, spermatogenesis is an ongoing process throughout most of the male's life. Consequently, the number of germ-line divisions is much higher in males than females and this sex gap widens with age [reviewed in 37,38]. More replications augment the chance for spontaneous germ-line mutations that arise during the mitotic phase of spermatogenesis. Transmitted to their offspring by older fathers, these germ-line mutations might cause very rare diseases such as achondroplasia and craniosynostosis (Apert's, Crouzon's and Pfeiffer's syndromes), as well as other disorders reviewed in [37–40]. However, with advancing age, the numerous replications of the male germ-line also may exert a powerful selection pressure that yields stem cells, which survive the impact of aging. This has been shown in aging male Drosophila melanogaster in which the population of germ-line stem cells is only a subset of the original population at a younger age [41].

Oxidative stress inflicts considerable damage on hematopoietic stem cells [42], and, in principle, this may apply to other stem cells. As oxidative stress heightens DNA damage and telomere loss [43,44], it may be a key determinant in aging-related diseases [45]. Thus, sperm with longer telomeres might arise from a subset of germ-line stem cells that either sustained less aging-related oxidative stress—perhaps because of increased resistance to its action—or underwent fewer replications prior to meiosis. Elongation of telomere length might also arise from epigenetic processes that take place in stem cells of the germ-line as men get older [46]. The genetic makeup of these sperm (with or without DNA mutations) would then be transmitted across generations, endowing some offspring with longer LTL.

Telomere length is highly variable among chromosomes [47]. Analyses of single chromosomes in somatic cells of parents and their offspring and in twins indicate that the inheritance of telomere length is allele specific [48–50]. This suggests that telomere length is determined in the zygote, with no apparent role of telomerase to match the lengths of homologous telomeres [reviewed in 51]. As the paternal age effect on LTL has not been examined in newborns, we do not know whether it is mediated by endowing the offspring with longer telomeres at birth or by attenuating the rate of telomere shortening afterwards.

In conclusion, offspring's LTL positively correlates with paternal age. A possible explanation for this phenomenon is the presence in older men of a subset of sperm with longer telomeres. The potential impact of paternal age on aging-related diseases and longevity in offspring is of particular relevance because offspring of older fathers comprise an increasing proportion of society [52,53] and as the dependency of offspring's LTL on paternal age does not display a paternal age threshold.

Finally, the paternal age effect joins a growing list of factors that impact leukocyte telomere biology in humans, underscoring the complex nature of LTL. Deciphering the genetic, epigenetic and environmental factors that account for inter-individual variation in LTL may provide considerable insight into the biology of human aging and aging-related diseases.

## Methods

### Subjects.

To be included in the first phase of the study, each individual (offspring) had to meet two criteria: a) the offspring's LTL was available from ongoing investigations, and b) the offspring's age (at blood collection) and parents' ages at the time of the offspring's birth were available.

For the first phase, we studied 235 males and 197 females from the Framingham Offspring, 355 males and 492 females from the NHLBI-Heart, 44 male and 88 female twins from the Danish Twins, and 1,954 females from UK Twins. Data on individuals and their partner's occupation were available from UK Twins to ascertain SES using the UK registrar general's classification [54]. No comparable SES information was available for the other cohorts.

The Framingham Heart Study includes the original cohort and a second cohort, the Framingham Offspring [55]. LTLs of the Framingham Offspring were from blood collected at exam 6 (1995–1998). LTLs from the NHLBI-Heart were from blood collected at the follow-up exams of the original pedigrees from 2002–2003 [56]. The Longitudinal Study of Aging Danish Twins started in 1995 and the LTLs of the Danish Twins were from blood collected during the 1997 survey [57]. LTLs from UK Twins [58] were from blood collected in 2001–2004.

For the second phase, we recruited sperm donors from the staff, student body, and faculty of the UMDNJ, NJMS. These were all healthy Caucasian men. For the Southern blot analysis, we studied 8 young (18–19 years) and 8 older (50–59 years) donors. For the Q-FISH analysis, we measured telomere signal intensity in X and Y sperm from 8 individuals (age 21–60 years), and in sperm sorted based on MMP from 5 young (20–30 years), and 5 older (56–65 years) donors. For the flow-FISH analysis, we studied sperm from 11 young (22–28 years) and 9 older (51–65 years) donors. Older and young donors provided their sperm on the same day, so that sperm from the two groups were processed in parallel.

### Telomere parameter measurements.

For the first phase, LTL was determined from Southern blots of the TRFL, as described before [7]. For the second phase, we measured sperm telomere parameters by Southern blots of TRFL (Figure 2), Q-FISH (Figure 3), and flow-FISH (Figure 4). We used chicken red cell nuclei (CRBCN) from a single stock as a constant reference for telomere signal for the Q-FISH (Figure 3) and flow-FISH (Figure 4) analyses.

Sperm samples were processed within two hours of ejaculation. They were maintained at room temperature for 30 min. Samples were processed with Viscolytic System at 35 °C for 20 min and filtered through SpermPrep II Sephadex columns (ZDL) for DNA isolation in preparation for TRFL analysis and Q-FISH analysis of telomeres in sperm subsets based on MMP. Samples were filtered through double layers of Miracloth (Calbiochem), sonicated for 10 sec × 3 on ice, and collected by centrifugation (10 min, 600 g) for flow-FISH analysis and Q-FISH analysis of X and Y sperm.

For the Q-FISH of telomere signals in X and Y sperm, the isolated sperm were re-suspended in PBS, treated with Fix & Perm Reagent A and Fix & Perm Reagent B (Caltag Lab) plus 10 mmol/L DTT and 0.05 μg/μL heparin for 15 min at room temp. Sperm were cytospinned onto slide glasses, air dried and kept at −20 °C until use. Q-FISH: Slides were treated with 1 mg/mL pepsin plus 10 mmol/L DTT and 0.05 μg/μL heparin for 10 min at 37 °C and dehydrated by a series of ethanol incubation (70, 90, and 100%, 1 min each) and air dried. Slides were hybridized in 70% formamide, 1% blocking reagent (Roche, Cat#1096176), 10 mmol/L Tris; pH 7.2, 2 ng/μL cy-5 labeled (CCCTAA)_3_ PNA probe, FITC labeled PNA probe for the Y chromosome (Dako) and rhodamine labeled PNA probe for the X chromosome (a gift from PerSeptive Biosystems). Preparations were denatured at 75 °C for 10 min. Slides were kept at room temp for 2 hrs, washed with 70% formamide, 10 mmol/L Tris; pH 7.2 for 15 min at room temp 2 times and washed with PBS + 0.025 % tween for 5 min at room temp 3 times and counterstained with 200 mg/mL DAPI. Telomere signals were captured and quantified by fluorescence microscope (Zeiss), Metafar (Metasystem) from 100–200 X sperm and similar numbers of Y sperm on each slide.

For the Q-FISH of telomere signals in unsorted sperm and sperm sorted based on DiOC_6_(3) stain, sperm were suspended in PBS and stained with DiOC_6_(3) at 37 °C for 20 min and 2.5 μg/mL propidium iodide (PI) added. They were sorted by FACS Vantage SE with FACS DiVa option (BD Biosciences) into 3 fractions (PI positive, PI negative with low DiOC_6_(3) staining, and PI negative with high DiOC_6_(3) staining [36]. Slides were then prepared as above for telomere analysis. A reference consisting of telomere signals from CRBCN was smeared onto the slide glass next to sperm cells and air dried. Telomere signals from sperm and CRBCN were captured and quantified as above. Telomere length in relative units was expressed by the intensity ratio of telomere signal from sperm/ CRBCN.

For flow-FISH, isolated sperm were hybridized in 70% formamide, 1% blocking reagent, 10 mmol/L Tris; pH 7.2, 2 ng/μL cy-5 labeled (CCCTAA)_3_ PNA probe and denatured at 75 °C for 10 min. CRBCN were hybridized in 0.2 ng/μL cy-5 labeled (CCCTAA)_3_ PNA probe plus 1.8 ng/μL unlabeled (CCCTAA)_3_ PNA probe. Sperm were kept at room temp over night and washed with 70% formamide, 10 mmol/L Tris; pH 7.2, 0.1% BSA, 0.1% tween 20 for 15 min at room temp (x 4) and 0.15 mol/L NaCl, 50 mmol/L Tris pH 7.5, 0.1% BSA, 0.1% tween 20 for 5 min (x 4). Sperm and CRBCN were re-suspended in PBS for FACS analysis. The acquisition of light scatter and fluorescence signals were done on a FACSCalibur flow cytometer (BD Biosciences) and the analysis performed with CellQuest software (BD Biosciences). To correct for daily shifts in the linearity of the flow cytometer and fluctuations in laser intensity and alignment, SPHERO Rainbow Calibration particles (Spherotech) were acquired at the beginning of each experiment.

A total of 10,000 events (sperm) were acquired for each specimen analyzed. The fluorescence of gated events was typically analyzed on a linear scale. CRBCN and sperm were distinguished based on their forward and side angle scatter properties.

All telomere measurements for both phases of the study were performed without knowledge about the donors of blood or sperm.

### Statistical analysis.

For the first phase, means and standard deviation (SD) for continuous variables or proportions for categorical variables were computed for all study variables. To evaluate the relationships between LTL, age, parent's age at birth of an offspring, and covariates, we performed correlation and linear regression analyses. In the regression analyses we used LTL as a dependent and parents' ages as primary predictor variables with age, BMI, cigarette smoking and SES, if available, as covariates. For the NHLBI-Heart, UK Twins and Danish Twins data, non-independence of family members or twins and the fact that they shared the same father was adjusted for by regression clustering by family number. All analyses were performed for males and females, separately. SAS (SAS Institute) was used to analyze the Framingham Offspring (version 9.1) and NHLBI – Heart (version 8.2) data. Stata software was used for UK Twins (version 9) and Danish Twins (version 9.2) data. We note that beta coefficients are presented in the tables and correlation coefficients are presented in Figure 1.

For the second phase, we used Student t test for data displayed in Figures 2A and 4A and repeated measures ANOVA for data displayed in Figure 3B. For the flow-Fish analysis (Figure 4B), we also utilized normal probability plots and the Fisher exact test comparing two proportions to examine regions over which there was a greater proportion of longer telomeres in older subjects. Vertical bars in figures denote SE.

### Informed consent.

The Boston Medical Center and the New Jersey Medical School Institutional Review Boards, the review boards at each NHLBI FHS center, the Scientific-Ethical Committee for Vejle and Funen Counties, and the St. Thomas' Hospital Research Ethics Committee approved the studies. All participants gave written informed consent.

## Supporting Information

Table S1 Characteristics of the Framingham Offspring Participants with LTL, Parent's Age at Offspring's Birth, and Covariate data (58 KB DOC)

Table S2 Characteristics of the NLHBI-Heart Participants with LTL, Parent's Age at Offspring's Birth, and Covariate Data (56 KB DOC)

Table S3 Characteristics of the Danish Twins Participants with LTL, Parent's Age at Offspring's Birth, and Covariate Data (46 KB DOC)

Table S4 Characteristics of the Whole Cohort and a Sub-Sample with Full Covariate Data of the UK Twins (62 KB DOC)
