# Supplementary material for: Offspring's Leukocyte Telomere Length, Paternal Age, and Telomere Elongation in Sperm
Source: PLoS Genet. 2008 Feb 15;4(2):e37. doi: 10.1371/journal.pgen.0040037 (PMC2242810; doi:10.1371/journal.pgen.0040037)
Supplement: Supplementary file 1 [file pgen.0040037.st001.doc]

**Table 1S:** Characteristics of the Framingham Offspring participants with LTL, parent’s age at offspring’s birth and covariate data

|  | **Characteristic** | **Mean** | **Std Dev** | **Minimum** | Maximum |
| --- | --- | --- | --- | --- | --- |
| **Male** | Son's age (years) | 56.9 | 9.1 | 37 | 80 |
| (n=235) | LTL (kb) | 6.98 | 0.56 | 5.70 | 8.52 |
|  | Father's age (years) | 32.2 | 5.7 | 17 | 55 |
|  | Mother's age (years) | 29.5 | 5.2 | 17 | 43 |
|  | BMI (kg/m^2^) | 28.4 | 4.5 | 18 | 47 |
|  | Smoking, n (%) |  |  |  |  |
|  | Never | 89 (38) |  |  |  |
|  | Past | 112 (48) |  |  |  |
|  | Current | 34 (14) |  |  |  |
|  |  |  |  |  |  |
| **Female** | Daughter's age (years) | 56.7 | 9.2 | 37 | 77 |
| (n=197) | LTL (kb) | 7.13 | 0.60 | 5.87 | 8.64 |
|  | Father's age (years) | 33.1 | 6.0 | 21 | 52 |
|  | Mother's age (years) | 30.1 | 5.5 | 17 | 43 |
|  | BMI (kg/m^2^) | 27.5 | 5.8 | 17.2 | 49.6 |
|  | Smoking, n (%) |  |  |  |  |
|  | Never | 75 (38) |  |  |  |
|  | Past | 87 (44) |  |  |  |
|  | Current | 35 (18) |  |  |  |
