# Supplementary material for: Offspring's Leukocyte Telomere Length, Paternal Age, and Telomere Elongation in Sperm
Source: PLoS Genet. 2008 Feb 15;4(2):e37. doi: 10.1371/journal.pgen.0040037 (PMC2242810; doi:10.1371/journal.pgen.0040037)
Supplement: Supplementary file 2 [file pgen.0040037.st002.doc]

**Table 2S:** Characteristics of the NLHBI-Heart participants with LTL, parent’s age at offspring’s birth and covariate data

|  | Characteristic | **Mean** | **Std Dev** | **Minimum** | **Maximum** |
| --- | --- | --- | --- | --- | --- |
|  |  |  |  |  |  |
| Male | Son's age (years) | 56.1 | 13.3 | 32 | 86 |
| (n=355) | LTL (kb) | 6.71 | 0.63 | 5.14 | 8.91 |
|  | Father's age (years) | 31.7 | 7.7 | 18 | 68 |
|  | Mother's age (years) | 28.3 | 6.5 | 17 | 46 |
|  | BMI (kg/m^2^) | 28.8 | 4.5 | 19.7 | 44.9 |
|  | Smoking, n (%) |  |  |  |  |
|  | Never | 152 (43) |  |  |  |
|  | Ever | 203 (57) |  |  |  |
|  |  |  |  |  |  |
| **Female** | Daughter's age (years) | 57.6 | 12.9 | 32 | 84 |
| (n=492) | LTL (kb) | 6.80 | 0.68 | 4.51 | 8.73 |
|  | Father's age (years) | 31.3 | 6.8 | 19 | 56 |
|  | Mother's age (years) | 28.4 | 6.1 | 17 | 53 |
|  | BMI (kg/m^2^) | 28.3 | 5.9 | 18.4 | 57.2 |
|  | Smoking, n (%) |  |  |  |  |
|  | Never | 259 (53) |  |  |  |
|  | Ever | 233 (47) |  |  |  |
