# Supplementary material for: Offspring's Leukocyte Telomere Length, Paternal Age, and Telomere Elongation in Sperm
Source: PLoS Genet. 2008 Feb 15;4(2):e37. doi: 10.1371/journal.pgen.0040037 (PMC2242810; doi:10.1371/journal.pgen.0040037)
Supplement: Supplementary file 3 [file pgen.0040037.st003.doc]

**Table 3S:** Characteristics of the Danish Twins participants with LTL, parent’s age at offspring’s birth and covariate data

|  | Characteristic | **Mean** | **Std Dev** | **Minimum** | **Maximum** |
| --- | --- | --- | --- | --- | --- |
|  |  |  |  |  |  |
| **Male** | Son's age (years) | 76.8 | 3.2 | 74 | 85 |
| (n=44) | LTL (kb) | 5.81 | 0.55 | 4.85 | 7.28 |
|  | Father's age (years) | 34.8 | 9.1 | 18 | 52 |
|  | Mother's age (years) | 31.5 | 6.5 | 19 | 46 |
|  |  |  |  |  |  |
| **Female** | Daughter's age (years) | 77.5 | 4.4 | 73 | 94 |
| (n=88) | LTL (kb) | 5.91 | 0.63 | 3.94 | 7.38 |
|  | Father's age (years) | 34.2 | 6.6 | 23 | 61 |
|  | Mother's age (years) | 30.8 | 4.3 | 23 | 42 |
