# Supplementary material for: Offspring's Leukocyte Telomere Length, Paternal Age, and Telomere Elongation in Sperm
Source: PLoS Genet. 2008 Feb 15;4(2):e37. doi: 10.1371/journal.pgen.0040037 (PMC2242810; doi:10.1371/journal.pgen.0040037)
Supplement: Supplementary file 4 [file pgen.0040037.st004.doc]

**Table 4S:** Characteristics of the whole cohort and a sub-sample with full covariate data of the UK Twins

|  | **Whole Sample** | | | | **Sub-sample** | | | |
| --- | --- | --- | --- | --- | --- | --- | --- | --- |
| **Characteristic** | N | **Mean** | **SD** | **Range** | **N** | **Mean** | **SD** | **Range** |
|  |  |  |  |  |  |  |  |  |
| **Age (years)** | 1954 | 48.0 | 12.7 | 18 -79 | 1000 | 45.2 | 10.3 | 18 - 72 |
|  |  |  |  |  |  |  |  |  |
| **LTL (kb)** | 1954 | 7.06 | 0.68 | 5.15 - 9.36 | 1000 | 7.12 | 0.68 | 5.15 – 9.33 |
|  |  |  |  |  |  |  |  |  |
| **Father’s Age**  **(years)** | 1736 | 33.1 | 6.3 | 18 – 58 | 1000 | 33.3 | 6.2 | 18 – 58 |
|  |  |  |  |  |  |  |  |  |
| **Mother’s Age**  **(years)** | 1766 | 30.4 | 5.2 | 16 – 44 | 1000 | 30.5 | 5.1 | 16 – 44 |
|  |  |  |  |  |  |  |  |  |
| **BMI (kg/m2)** | 1566 | 25.3 | 4.6 | 11.7 – 52.7 | 1000 | 25.0 | 4.5 | 14.47 – 48.06 |
|  |  |  |  |  |  |  |  |  |
| **Smoking** | 1586 | 0 = 52.65%  1 = 17.72%  2 = 29.63% | n/a | n/a | 1000 | 0=52.85% 1= 18.38%  2= 28.77% | n/a | n/a |
|  |  |  |  |  |  |  |  |  |
| **SES** | 1126 | 1= 24.51% 2= 38.54% 3= 17.14%  4 = 6.39%  5 = 13.42% | n/a | n/a | 1000 | 1= 24.2%  2= 39.6%  3= 16.4%  4= 6.4%  5= 13.5% | n/a | n/a |

* For smoking, 0= never smoked, 1= ever smoked, 2 = current smoker

** For socio-economic status (SES), 1 = professional occupations, 2 = managers & administrators and associate professional & technical workers, 3 = clerical and secretarial non-manual skilled occupations, 4 = crafts & manual related skilled occupations, 5 = partly skilled and unskilled occupations
